# Unravelling the hidden link of lithium halides and application in the synthesis of organocuprates

**DOI:** 10.1038/ncomms14794

**Published:** 2017-03-16

**Authors:** Hong Yi, Dali Yang, Jie Xin, Xiaotian Qi, Yu Lan, Yi Deng, Chih-Wen Pao, Jyh-Fu Lee, Aiwen Lei

**Affiliations:** 1 College of Chemistry and Molecular Sciences, The Institute for Advanced Studies (IAS), Wuhan University, Wuhan, Hubei 430072, China; 2 School of Chemistry and Chemical Engineering, Chongqing University, Chongqing 400030, China; 3 National Synchrotron Radiation Research Center, Hsinchu 30076, Taiwan; 4 State Key Laboratory of Organometallic Chemistry, Shanghai Institute of Organic Chemistry, Chinese Academy of Sciences, Shanghai 200032, China

## Abstract

As a versatile metal, copper has demonstrated a wide application in acting as both organometallic reagent and catalyst. Organocuprates are among the most used organometallic reagents in the formation of new carbon–carbon bonds in organic synthesis. Therefore, revealing the real structures of organocuprates in solution is crucial to provide insights into the reactivity of organocuprates. Here we provide several important insights into organocuprate chemistry. The main finding contains the following aspects. The Cu(0) particles were detected via the reduction of Cu*X* by *n*BuLi or PhLi. The Cu(II) precursors Cu*X*_2_ (*X*=Cl, Br) could be used for the preparation of Gilman reagents. In addition, we provide direct evidence for the role and effect of Li*X* in organocuprate synthesis. Moreover, the EXAFS spectrum provides direct evidence for the exact structure of Li^+^ Cu*X*_2_^−^ ate complex in solution. This work not only sheds important light on the role of Li*X* in the formation of organocuprates but also reports two new routes for organocuprate synthesis.

## Abstract (web summary)

Organocopper species are widely used in synthetic chemistry. Here the authors study the structure of the anionic complex formed from copper salts and lithium halides, showing it to be a key intermediate in the formation of organocuprates, and also show that Cu(II) precursors can form Gilman reagents.

Since the pioneering work of Gilman *et al*.,^1^ organocuprates have been widely employed as organometallic reagents in organic synthesis (including conjugate additions, the opening of epoxides and cross-coupling reactions)^2^,^3^,^4^,^5^,^6^,^7^. In the textbook, organocuprates are usually prepared through transmetalation of lithium, magnesium or zinc organometallics with Cu(I) salts^8^. Different coordination environments always drastically affect the reactivity or stabilities of organocuprates^9^. Up to now, a lot of synthetic methodologies involving organocuprate reagents have been developed, while great uncertainty still exists in the related mechanism^10^. Although several important crystal structures of organocuprates were reported^11^,^12^,^13^,^14^, it should be noted that solid-state structures often reflect the most thermodynamically stable species and are not necessarily the same as in solution state. Besides, organocuprates can exhibit complex behaviour in solution, often existing as a number of different species in equilibrium, thus further complicating their characterization. For that reason, the structure of organocopper compounds in solution cannot be inferred directly from crystal structures and must be determined independently.

The structures of organocuprate reagents in an ethereal solution have received wide attention, because they are strongly relevant to reactivity in real reaction conditions^15^,^16^,^17^. Nuclear magnetic resonance^18^,^19^,^20^ and electrospray ionization–mass spectrometry^21^,^22^ served as powerful tools and have been widely used in determining the structures of organocuprates in solution. The linear bonding geometry of the C–Cu–C moiety in cuprates such as MeCu(CN)Li, Me_2_CuLi and Me_2_-CuLi_3_Li*X* (*X*=I, CN) has been well established. In 1996, Knochel and colleagues^23^,^24^ first introduced the extended X-ray absorption fine structure (EXAFS) to study the local structure of organocuprates from the reaction between CuCN and *n*BuLi. EXAFS spectroscopy provides a unique probe of the local structural environment of metal ions in non-crystalline systems^25^,^26^,^27^,^28^,^29^,^30^,^31^,^32^,^33^. The preliminary structure for lithium cyanocuprates based on EXAFS data has been elucidated. However, the role of cyanide and the difference between cyanide and other halide atoms still remain in debate^9^. Lipshutz *et al*.^21^ and Koszinowski and colleagues^34^ have pointed out the Li*X* could have a positive effect on the solubility of Cu*X* (*X*=I, Br, Cl, CN) independently. The electrospray ionization–mass spectrometry was used to study the structure of formed ate complex^21^,^34^. However, determination of the exact structure, the role for Li*X* and application in organocuprates have still been not well-studied up to date. We started our research by investigating the effect of anion on organocuprates preparation. Here we show the anion effect of different Cu(I) precursors on Gilman reagent preparation. The EXAFS reveals that the Li*X* (*X*=Br, Cl) serves as the hidden link for organocuprates preparation from unfavoured Cu*X*. A soluble cupric bromide anion intermediate is evidenced by EXAFS when adding LiBr to CuBr in tetrahydrofuran (THF). This Cu*X*_2_^−^ Li^+^ ate complex serves as a key intermediate in the generation of Gilman reagent (Fig. 1). In addition, we also shed two other important findings in this work. First, the detection of copper nanoparticles produced after the addition of *n*BuLi or PhLi to Cu*X*. Second, the Cu(II) precursors Cu*X*_2_ (*X*=Cl, Br) can be used for the preparation of Gilman reagents.

## Results

### Detection of Cu(0) particles via the reduction of Cu*X*

**Figure 2:**
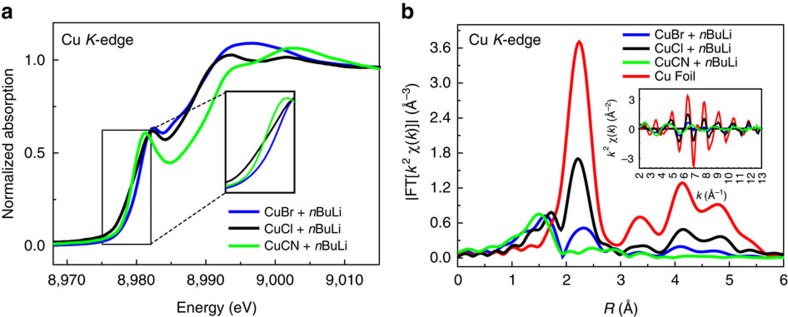
XAFS analysis of Cu*X* reduction. (**a**) XANES spectra of various cuprous salts with excess *n*BuLi in THF under −78 °C. (**b**) Comparison of FT magnitudes of *k*^2^-weighted EXAFS of various Cu(I) species mixed with excess *n*BuLi in THF under −78 °C. (3.0 Å^−1^<*k*<12.4 Å^−1^).

In organic synthesis, different Cu(I) precursors are always applied in different reaction systems^10^. Initially, we investigated different cuprous salts with excess *n*BuLi in THF under −78 °C for organocuprates synthesis. From X-ray absorption near-edge spectroscopy (XANES) spectra (Fig. 2a), we observed the difference of reactions from CuCN and Cu*X* (*X*=Br, Cl). In Fourier-transformed EXAFS spectra, an obvious copper nanoparticle feature at high shells in CuBr and CuCl complexes appeared (Fig. 2b). However, such peaks at 3.4, 4.1 and 4.8 Å were not detected in the CuCN system, which is accordance with previous literature^23^ that CuCN is a good precursor to Gilman reagent. In addition, such results also indicate that organocuprates made by CuBr or CuCl are very unstable to decompose into zero valance copper nanoparticle. Meanwhile, it seemed that the smaller the anion atom is, the more Cu(0) particle is formed.

**Figure 3:**
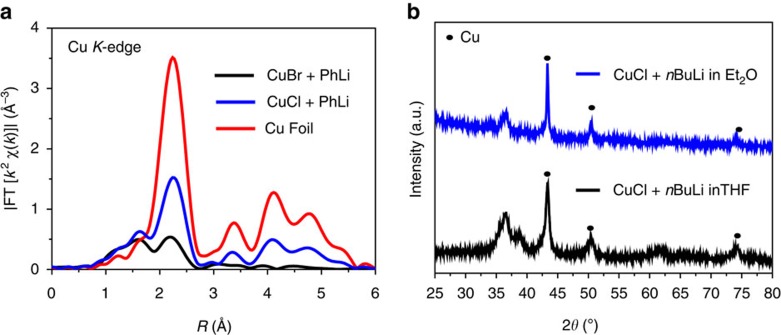
EXAFS and X-ray powder diffraction analysis. (**a**) EXAFS spectra of of CuBr and CuCl mixed with excess PhLi in THF under −78 °C. (**b**) X-ray powder diffraction experiments, blue line: CuCl+5.0 equiv *n*BuLi in Et_2_O, black line: CuCl+5.0 equiv *n*BuLi in THF.

Then, we also used X-ray absorption spectroscopy (XAS) to study the reaction between Cu*X* (*X*=Br, Cl) and PhLi. The XANES spectra were shown in Supplementary Fig. 1. From the EXAFS spectra in Fig. 3a, we found that the mixture of Cu (0) and Cu (I) species was formed when mixing CuBr or CuCl with PhLi. The CuCl was easier to be reduced to Cu(0) particle than CuBr by PhLi, which was consistent with the reaction with *n*BuLi. In addition, we also investigated the solvent effect on this process. We found that the reduction process was even faster when using ethyl ether (Et_2_O) as the solvent (Supplementary Figs 2 and 3). To further evidence the Cu(0) species and this reduction process, X-ray powder diffraction experiments were performed and the results are shown in Fig. 3b. The figure shows the main existence of Cu in the reaction between CuCl and *n*BuLi in THF or Et_2_O, in which the three peaks at 43.3°, 50.4° and 74.1° are corresponding to the (111), (200) and (220) planes of Cu (JCPDS number 04-0836), respectively.

### Reduction of Cu(II) precursors

**Figure 4:**
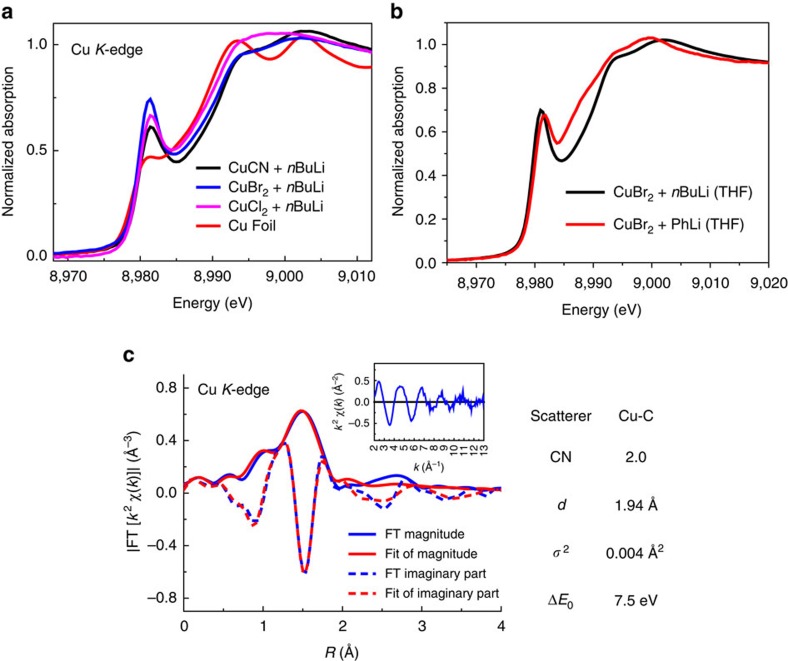
XAFS analysis of Cu*X*_2_ reduction. (**a**) XANES spectra of CuCN, CuBr_2_ and CuCl_2_ mixed with excess *n*BuLi in THF under −78 °C. (**b**) XANES spectra of CuBr_2_ mixed with excess PhLi in THF under −78 °C. (**c**) Fitting result for CuBr_2_+5.0 equiv *n*BuLi in THF solution (2.910 Å^−1^<*k*<11.472 Å^−1^ and 1.065 Å<*R*<2.127 Å).

As the *n*BuLi could serve as a reductant to reduce Cu*X* (*X*=Br, Cl) to Cu(0) species, we also employed EXAFS to investigate the reactions between Cu(II) salts and *n*BuLi. We are very excited to discover that instead of using Cu(I) as the starting reagent, more stable and cheaper Cu(II) halide salts could also be good Gilman reagent precursors in the presence of excess organolithium reagent. EXAFS provided us a direct view of these transformations. Compared with traditional Gilman reagent prepared from CuCN, we could see that in the presence of 5.0 equivalent of *n*BuLi in THF under −78 °C, both CuBr_2_ and CuCl_2_ were reduced into Cu(I) with edge energies about 8979.9 and 8980.0 eV, respectively, in the XANES spectra (Fig. 4a). The PhLi could also reduce CuBr_2_ to corresponding Cu(I) species (Fig. 4b). The fitting result further verified the existence of 2-coordinated [C-Cu(I)-C] short-range structure (Fig. 4c). Owing to the fact that Cu*X*_2_ is stable and easy to store, this method will be a new route for organocuprates preparation from Cu*X*_2_. We also applied this method to organic synthesis. When adding the electrophile such as benzyl bromide or (2-bromoethyl)benzene into the reaction system, the desired C–C bond formation was formed, which implied that this method for organocuprates could be used for cross-coupling reactions. The detail application of this method in complicated molecular synthesis is on the way in our lab.

### Role of lithium halides in the synthesis of organocuprates

**Figure 5:**
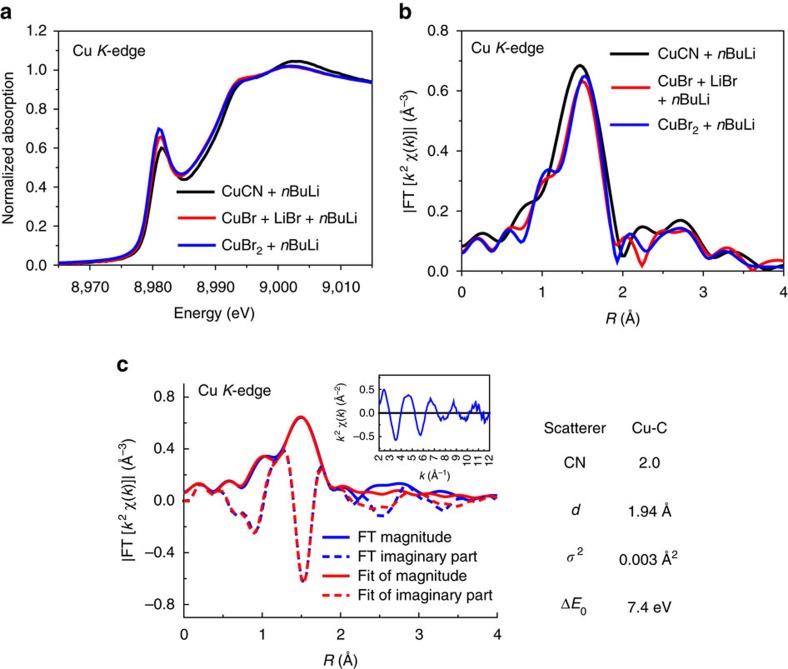
Role of lithium halides in the synthesis of organocuprates. (**a**) XANES spectra of CuCN (black), CuBr+1.0 eq LiBr (red) and CuBr_2_ (blue) with excess *n*BuLi in THF under −78 °C. (**b**) EXAFS spectra of CuCN (black), CuBr+1 eq LiBr (red) and CuBr_2_ (blue) with excess *n*BuLi in THF under −78 °C. (**c**) Fitting results of *R*-space *k*^2^-weighted EXAFS spectra of CuBr+1 equiv LiBr in 5.0 equiv ^*n*^BuLi THF solution (2.957 Å^−1^<*k*<11.145 Å^−1^ and 1.172 Å<*R*<1.914 Å).

The lithium salts may have an effect on the formation of organocuprates, which has been awaked by several groups^21^,^34^,^35^,^36^,^37^,^38^,^39^. Although knowing this phenomenon for a long time, determination of the exact structure, the role for Li*X* and application in organocuprates have still been not well researched. When one equivalent LiBr was added to the mixture of CuBr and 5 equivalent *n*BuLi, we obtained a similar XANES spectra of which obtained from CuCN and excess *n*BuLi under low temperature (Fig. 5a). The edge energy was determined as 8979.7 and 8979.9 eV, respectively. Furthermore, high shell features for copper nanoparticle disappeared in the EXAFS spectrum, which indicates the formation of relatively pure organocopper compound (Fig. 5b). The results of fitting show two carbon atoms at 1.94 Å around the copper atom equally (Fig. 5c). Thus, we developed a new method of preparing Gilman reagents using Cu*X* with the help of Li*X*, which is very similar to what we get from mostly used cuprous cyanide in traditional synthesis route.

**Figure 6:**
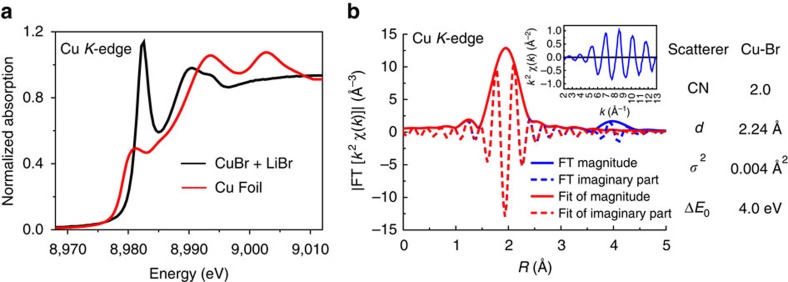
XAFS analysis of reaction between Cu*X* and Li*X*. (**a**) XANES spectra of CuBr+LiBr in THF species. (**b**) Fitting results of *k*^2^-weighted *R*-space EXAFS spectra of CuBr+1.0 equiv LiBr (2.890 Å^−1^<*k*<12.134 Å^−1^ and 1.487 Å<*R*<2.376 Å).

To further elucidate the role of Li*X* in preparing Gilman reagent, the mixture of LiBr and CuBr in THF was used for demonstration. As shown in Supplementary Fig. 4, the CuBr species alone look polymer-like and very insoluble in THF. In contrast, CuBr can be dissolved in THF with the aid of one equivalent quantity of LiBr. A green solution is quickly formed after adding 1 equivalent LiBr into the system. This result indicated that the CuBr has an interaction with LiBr and a new copper species is possibly formed. The interaction between CuBr and LiBr might be the key to stabilizing the Gilman reagents prepared through this method. Valance alternation in cuprous bromide was invisible in the presence of LiBr from XANES spectra (Fig. 6a, edge energy of 8980.9 eV). In addition, EXAFS fitting results indicate two bromine atoms coordinated to the copper(I) centre (Fig. 6b). Thus, we claim that this type of ate complex was a [Br-Cu-Br]^−^ Li^+^ anion. This [CuBr_2_]^−^ ate complex shows good solubility and serves as a key intermediate in the generation of Gilman reagent.

### Density functional theory calculations

**Figure 7:**
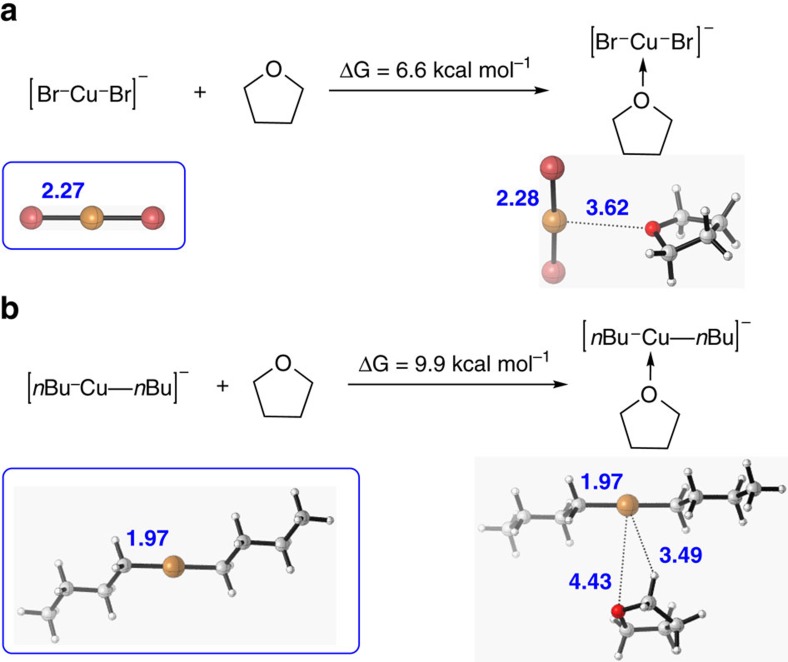
Density functional theory calculation. Density functional theory calculation for the coordination of THF to (**a**) [Br-Cu-Br]^−^ anion and (**b**) [*n*Bu-Cu-*n*Bu]^−^ anion.

Density functional theory calculation was also performed to provide support for the EXAFS fitting results. As shown in Fig. 7a, the coordination of THF to [Br-Cu-Br]^−^ anion is endergonic by 6.6 kcal mol^−1^. The optimized structure suggests that the Cu–O distance is 3.62 Å, which means the interaction between Cu and O is very weak. As previous reports, the monomer state of organocuprates was always present in more polar solvent such as THF. Thus, the monomer structure of organocuprate was calculated. Meanwhile, the coordination of THF to [*n*Bu-Cu-*n*Bu]^−^ anion is found to be endergonic by 9.9 kcal kcal mol^−1^ and the corresponding Cu–O distance is determined to be 4.43 Å (Fig. 7b). Thus, the coordination of THF to [Br-Cu-Br]^−^ and [^*n*^Bu-Cu-^*n*^Bu]^−^ anion are both energetically unfavourable^19^,^20^,^40^. Moreover, optimized structures reveal that the bond length of Cu–Br in [Br-Cu-Br]^−^ anion is 2.27 Å and the bond length of Cu–C in [*n*Bu-Cu-*n*Bu]^−^ anion is 1.97 Å, which are very close to that obtained from EXAFS (2.24 and 1.94 Å ). Consequently, the theoretical study and EXAFS fitting results have reached the same conclusion.

## Discussion

To sum up, we elucidate Li*X* (*X*=Br, Cl) as a hidden link in the preparation of organocuprate reagents from Cu*X* (*X*=Br, Cl) with a key intermediate Cu*X*_2_^−^ Li^+^ ate complex evidenced by EXAFS. Meanwhile, this Cu(I) ate complex can serve as a good precursor to prepare Gilman reagents following a tandem process. In addition, we also developed the organocuprate reagents synthesis from Cu(II) precursors. This discovery might help open a new perspective in understanding the organocopper chemistry and mechanisms of copper-catalysed reactions as well.

## Methods

### General information

X-ray absorption measurements were acquired in transmission mode at beamline 17C1 at National Synchrotron Radiation Research Center in Taiwan. A pure Cu foil spectrum (edge energy 8979, eV) was acquired simultaneously with each measurement for energy calibration. Multiple scans were taken to reduce the noise. The Supplementary Tables 1–3 revealed the detailed parameters of the XAFS spectral.

### Reaction system

Cu salt (0.5 mmol) was added to the schlenk tube cell in a glovebox beforehand. Then, 5.0 ml of THF was injected into the cell and the solution was stirred under N_2_ at −78 °C for 20 min. Subsequently, RLi (2.5 mmol) was added into the system and stirred for 30 min. As the last step, the liquid nitrogen was quickly added to reaction system, which would be frozen into solid immediately, and it was transferred into the XAFS cell with the protection of nitrogen gas.

### Detection system (beamline)

The detection system was cooled using cooled nitrogen gas. The Supplementary Fig. 5 was the picture of cell holder used in the beamline. This hold connected with a liquid nitrogen cooled gas stream. The Supplementary Fig. 6 showed our idea for low-temperature system. We used a gas stream passing through the liquid nitrogen Dewar to cool the system. The temperature could be controlled by the tuning of the flow rate. The Supplementary Fig. 7 was the whole picture of experimental set-up in beamline.

### Data availability

Data supporting the findings of this study are available within this article and its Supplementary Information file and from the corresponding authors on reasonable request.

## Additional information

**How to cite this article:** Yi, H. *et al*. Unravelling the hidden link of lithium halides and application in the synthesis of organocuprates. *Nat. Commun.* **8,** 14794 doi: 10.1038/ncomms14794 (2017).

**Publisher's note**: Springer Nature remains neutral with regard to jurisdictional claims in published maps and institutional affiliations.

## Supplementary Material

Supplementary Information Supplementary figures, supplementary tables, supplementary methods and supplementary references.

## Figures and Tables

**Figure 1:**
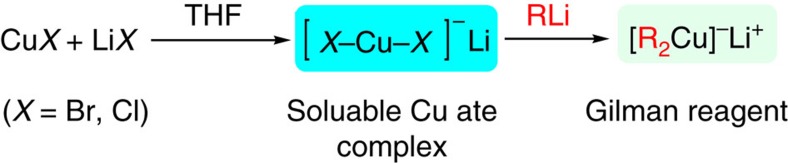
Hidden link of lithium halides. Scheme of the role of Li*X* (*X*=Br, Cl) and application in Gilman reagent preparation.
