# Supplementary material for: Unravelling the hidden link of lithium halides and application in the synthesis of organocuprates
Source: Nat Commun. 2017 Mar 16;8:14794. doi: 10.1038/ncomms14794 (PMC5357309; doi:10.1038/ncomms14794)
Supplement: Supplementary Information — Supplementary figures, supplementary tables, supplementary methods and supplementary references. [file ncomms14794-s1.pdf]

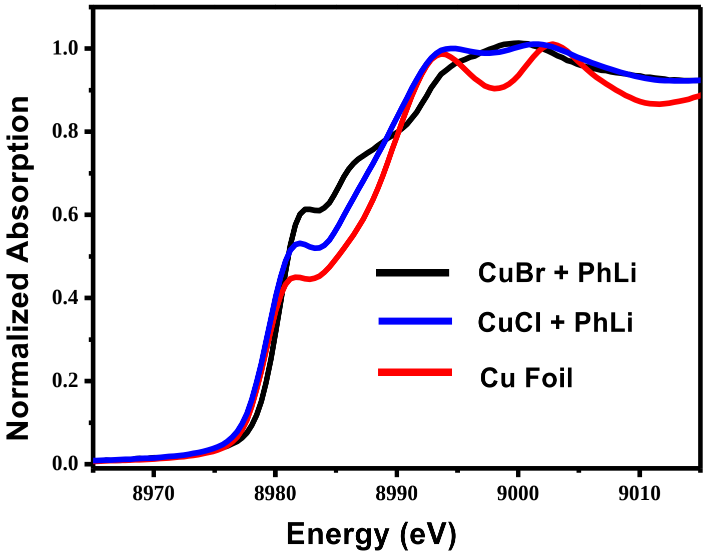

Supplementary Figure 1. XANES analysis. The XANES spectra of CuX species with PhLi

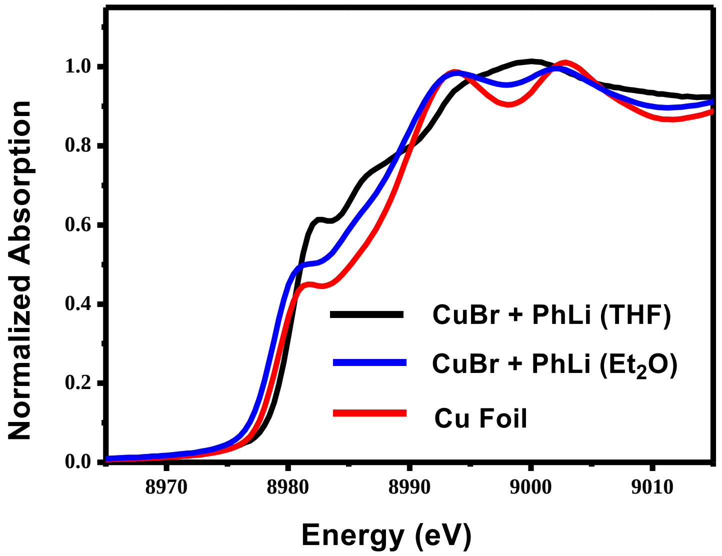

Supplementary Figure 2. XANES analysis of the solvent effect. The XANES spectra of CuX species with PhLi in THF or Et_2O

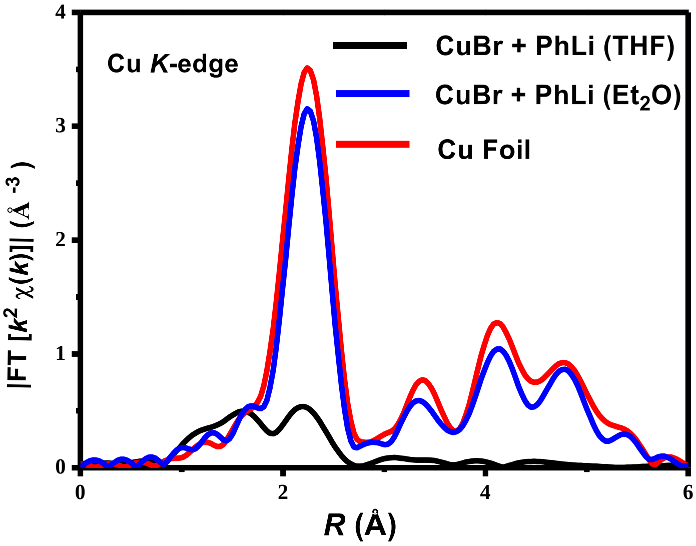

Supplementary Figure 3. EXAFS analysis. The EXAFS spectra of CuX species with PhLi in THF or Et_2O

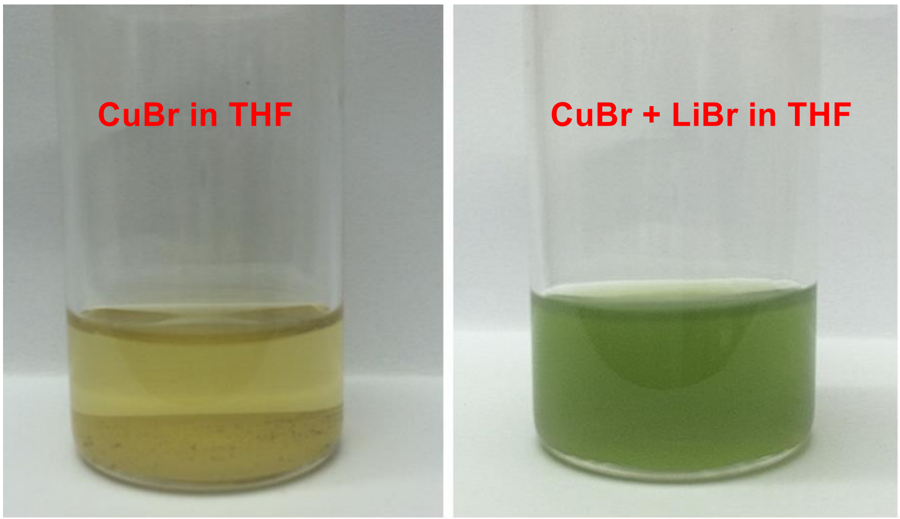

Supplementary Figure 4. Lithium halides effect. The Pictures of CuBr and CuBr + 1.0 eq LiBr in THF solution

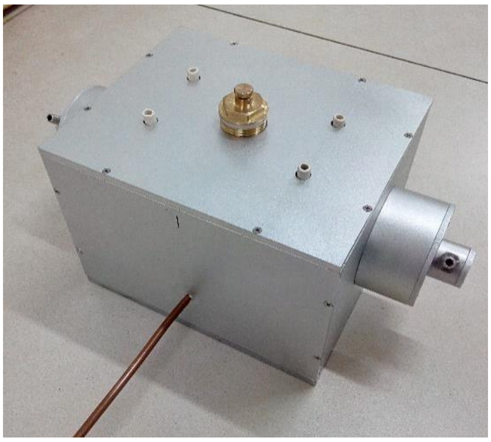

Supplementary Figure 5. The cell-holder for detection

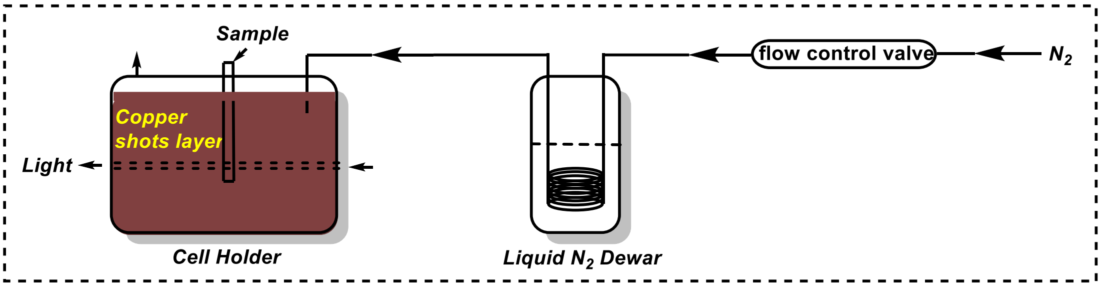

Supplementary Figure 6. The idea of low-temperature experiment using liquid N_2 Dewar

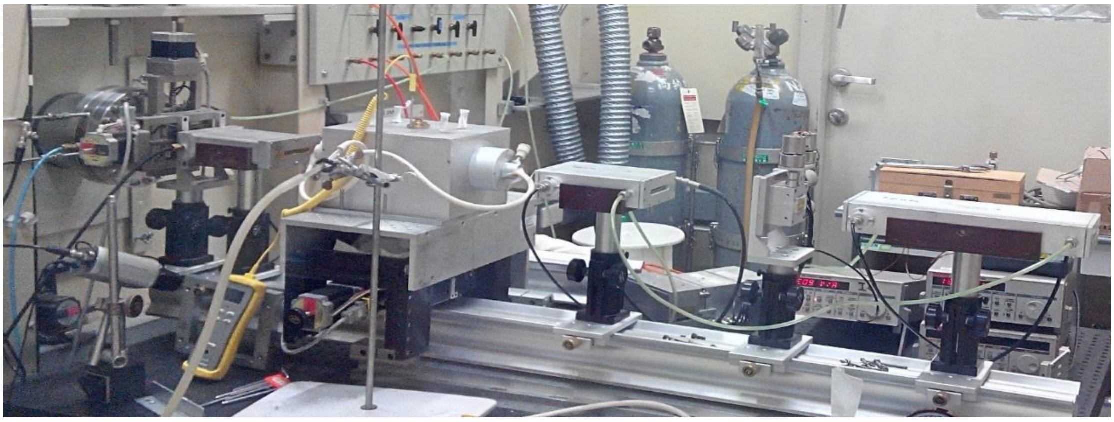

Supplementary Figure 7. The whole picture of the experimental set-up in the beamline

Supplementary Table 1. XAS Pre-Edge Energies and Edge Energy and Oxidation States for Copper Complexes

Sample	Edge Energy (eV)	Oxidation State	Comment
**Cu foil**	**8979.0**	**0**	**Reference**
**CuCl (Solid)**	**8981.9**	**+1**	**Reference**
**CuBr (Solid)**	**8981.4**	**+1**	**Reference**
**CuCN (Solid)**	**8980.3**	**+1**	**Reference**
**$CuCl_2$ (solid)**	**8978.0 (pre-edge)**	**+2**	**Reference**
**$CuBr_2$ (solid)**	**8976.3 (pre-edge)**	**+2**	**Reference**
**CuBr + *n*BuLi**	**-**	**+1/+0**	
**CuCl + *n*BuLi**	**-**	**+1/+0**	
**CuBr + PhLi**	**-**	**+1/+0**	
**CuCl + PhLi**	**-**	**+1/+0**	
**CuCN + *n*-BuLi**	**8979.6**	**+1**	
**CuBr2 + *n*-BuLi**	**8979.3**	**+1**	
**CuCl2 + *n*-BuLi**	**8980.1**	**+1**	
**CuBr2 + PhLi**	**8979.8**	**+1**	
**CuBr + LiBr + *n*-BuLi**	**8979.8**	**+1**	
**CuBr + LiBr**	**8981.4**	**+1**	

Supplementary Table 2. Structural Parameters of XAS Standard Samples

Sample	Scatterer	Coordination number	Bond distance (Å)	Reference
**Cu foil**	**Cu-Cu**	**12**	**2.54**	*(4)*
**CuCl (Solid)**	**Cu-Cl**	**4.0**	**2.30**	(5)
**CuBr (Solid)**	**Cu-Br**	**4.0**	**2.40**	(6)
**$CuCl_2$ (solid)**	**Cu-Cl**	**4.0**	**2.28**	(5)
**$CuBr_2$ (solid)**	**Cu-Br**	**4.0**	**2.40**	(7)

Supplementary Table 3. EXAFS First Shell Fitting Results

Sample	Scatterer	Amp (amplitude reduction factor)	Coordination number	Bond distance (Å)	σ^2 (Å^2)	ΔE_0 (eV)
CuCN (solid)	Cu-C<br>Cu-N<br>Cu-Cu	0.874<br>0.874<br>0.874	2.0<br>2.0<br>2.0	1.88<br>2.99<br>4.86	0.001<br>0.004<br>0.013	11.77<br>4.57<br>-1.83
CuCN + ***n*-BuLi** in THF	Cu-C	0.975	2.0	1.94	0.001	7.40
CuBr2 + ***n*-BuLi** in THF	Cu-C	0.875	2.0	1.94	0.004	7.50
CuBr + LiBr + ***n*-BuLi** in THF	Cu-C	0.875	2.0	1.94	0.003	7.4
CuBr + LiBr in THF	Cu-Br	0.968	2.0	2.24	0.004	4.0

Due to the lack of standard compounds from which the amplitude reduction factor can be extracted, we instead employed a theoretical model generated by the software of Feff 8. At the beginning of EXAFS data fitting, the coordination number was set to a positive integer value (1, 2, or other larger values) and let the amplitude reduction factor be free to change. Then, we would try different integer coordination numbers to fit the EXAFS data and see which value will yield an amplitude reduction factor between 0.8 and 1.0 (the commonly accepted range).

Supplementary Methods

XANES and EXAFS Data Collection and Analysis

1 General Information

X-ray absorption measurements were acquired in transmission mode at beamline 17C1[1] at National Synchrotron Radiation Research Center (NSSRC) in Taiwan. A pure Cu foil spectrum (edge energy 8979 eV) was acquired simultaneously with each measurement for energy calibration. Multiple scans were taken to reduce the noise.

All solution samples were placed in a sample holder (the XAS solution cell) made of PEEK (polyether ether ketone) equipped with a screw top and O-ring fitting to prevent exposure to air and water.[2] For solution samples, the Cu concentration was adjusted to be 0.05 - 0.1 M with a path length of 3.5 mm. The sample holder was placed in a quartz tube (1–in. OD, 10–in. length) sealed with Kapton windows by two Ultra-Torr fittings and then used for transmission mode measurement.

The edge energy of the X-Ray absorption near edge structure (XANES) spectrum was determined from the inflection point of the edge. The data procedures were carried out using the Athena software package using standard methods.[3] Standard procedures based on Artemis software (Demeter 0.9.20) were used to extract the extended X-ray absorption fine structure (EXAFS) data. The coordination parameters were obtained by the least square fit in R-space of the nearest neighbor, k^2 weighted Fourier transform data.

The X-ray diffraction (XRD) pattern was recorded by X-ray powde diffraction (XRD, PANalytical X-Pert Pro with Cu Kα radiation, λ=1.5406Å, Holland).

2. Experimental Details

2.1 CuX reduction process

2.1.1 CuX reduction by n-BuLi

CuBr / *n*-BuLi in THF solution: CuBr (0.5 mmol, 71.5 mg) was added to the XAS solution

cell in a glovebox beforehand. Then, 5.0 mL of THF was injected into the cell and the solution was stirred under N_2 at – 78 °C for 20 minutes. Then *n*-BuLi (2.5 mmol) was added into the system and stirred for 30 mins. XAFS spectrum was measured at -78 °C.

CuCl / *n*-BuLi in THF solution: CuCl (0.5 mmol, 49.75 mg) was added to the XAS solution cell in a glovebox beforehand. Then, 5.0 mL of THF was injected into the cell and the solution was stirred under N_2 at – 78 °C for 20 minutes. Then *n*-BuLi (2.5 mmol) was added into the system and stirred for 30 mins. XAFS spectrum was measured at -78 °C.

CuCN / *n*-BuLi in THF solution: CuCN (0.5 mmol, 45.0 mg) was added to the XAS solution cell in a glovebox beforehand. Then, 5.0 mL of THF was injected into the cell and the solution was stirred under N_2 at – 78 °C for 20 minutes. Then *n*-BuLi (2.5 mmol) was added into the system and stirred for 30 mins. XAFS spectrum was measured at -78 °C.

2.1.2 CuX reduction by PhLi

CuBr / PhLi in THF solution: CuBr (0.5 mmol, 71.5 mg) was added to the XAS solution cell in a glovebox beforehand. Then, 5.0 mL of THF was injected into the cell and the solution was stirred under N_2 at – 78 °C for 20 minutes. Then PhLi (2.5 mmol) was added into the system and stirred for 30 mins. XAFS spectrum was measured at -78 °C.

CuCl / PhLi in THF solution: CuCl (0.5 mmol, 49.75 mg) was added to the XAS solution cell in a glovebox beforehand. Then, 5.0 mL of THF was injected into the cell and the solution was stirred under N_2 at – 78 °C for 20 minutes. Then PhLi (2.5 mmol) was added into the system and stirred for 30 mins. XAFS spectrum was measured at -78 °C.

2.1.3 Solvent effect on CuX reduction process

CuBr / PhLi in Et_2O solution: CuBr (0.5 mmol, 71.5 mg) was added to the XAS solution cell in a glovebox beforehand. Then, 5.0 mL of THF was injected into the cell and the solution was stirred under N_2 at – 78 °C for 20 minutes. Then PhLi (2.5 mmol) was added into the system and

stirred for 30 mins. XAFS spectrum was measured at -78 °C.

2.1.4 General procedure for XRD experiment

CuCl / *n*-BuLi in THF solution: CuCl (0.5 mmol, 49.75 mg) was added to the XAS solution cell in a glovebox beforehand. Then, 5.0 mL of THF was injected into the cell and the solution was stirred under N_2 at – 78 °C for 20 minutes. Then *n*-BuLi (2.5 mmol) was added into the system and stirred for 30 mins. Prior to the XRD experiment, the product was centrifuged and washed with ethanol/water several times and finally dried in vacuum over at 60 °C for 24 h.

CuCl / *n*-BuLi in Et_2O solution: CuCl (0.5 mmol, 49.75 mg) was added to the XAS solution cell in a glovebox beforehand. Then, 5.0 mL of Et_2O was injected into the cell and the solution was stirred under N_2 at – 78 °C for 20 minutes. Then *n*-BuLi (2.5 mmol) was added into the system and stirred for 30 mins. Prior to the XRD experiment, the product was centrifuged and washed with ethanol/water several times and finally dried in vacuum over at 60 °C for 24 h.

2.2 CuX_2 reduction process by *n*-BuLi or PhLi

2.2.1 CuX_2 reduction by n-BuLi

$CuBr_2$ / *n*-BuLi in THF solution: $CuBr_2$ (0.5 mmol, 111.5 mg) was added to the XAS solution cell in a glovebox beforehand. Then, 5.0 mL of THF was injected into the cell and the solution was stirred under N_2 at – 78 °C for 20 minutes. Then *n*-BuLi (2.5 mmol) was added into the system and stirred for 30 mins. XAFS spectrum was measured at -78 °C.

$CuCl_2$ / *n*-BuLi in THF solution: $CuCl_2$ (0.5 mmol, 67.5 mg) was added to the XAS solution cell in a glovebox beforehand. Then, 5.0 mL of THF was injected into the cell and the solution was stirred under N_2 at – 78 °C for 20 minutes. Then *n*-BuLi (2.5 mmol) was added into the system and stirred for 30 mins. XAFS spectrum was measured at -78 °C.

$CuBr_2$ / PhLi in THF solution: $CuBr_2$ (0.5 mmol, 111.5 mg) was added to the XAS solution cell in a glovebox beforehand. Then, 5.0 mL of THF was injected into the cell and the solution was stirred under N_2 at – 78 °C for 20 minutes. Then PhLi (2.5 mmol) was added into the system and

stirred for 30 mins. XAFS spectrum was measured at -78 °C.

2.2.2 General procedure for organic synthesis

Step One: $CuBr_2$ / *n*-BuLi in THF solution: $CuBr_2$ (0.5 mmol, 111.5 mg) was added to the XAS solution cell in a glovebox beforehand. Then, 5.0 mL of THF was injected into the cell and the solution was stirred under N_2 at – 78 °C for 20 minutes. Then *n*-BuLi (2.5 mmol) was added into the system and stirred for 30 mins.

Step Two: Then 0.25 mmol electrophile such as benzyl bromide or (2-bromoethyl)benzene into the reaction system. The reactions were monitored by GC-MS. The C-C bond formation products were detected by GC-MS

2.3 CuX + LiBr by n-BuLi

CuBr + LiBr / *n*-BuLi in THF solution: CuBr (0.5 mmol, 71.5 mg), LiBr (0.5 mmol, 43.5 mg) was added to the XAS solution cell in a glovebox beforehand. Then, 5.0 mL of THF was injected into the cell and the solution was stirred under N_2 at – 78 °C for 20 minutes. Then *n*-BuLi (2.5 mmol) was added into the system and stirred for 30 mins. XAFS spectrum was measured at -78 °C.

2.4 CuBr + LiBr by XAFS

CuBr + LiBr in THF solution: CuBr (0.5 mmol, 71.5 mg), LiBr (0.5 mmol, 43.5 mg) was added to the XAS solution cell in a glovebox beforehand. Then, 5.0 mL of THF was injected into the cell and the solution was stirred under N_2 at room temperature for 20 minutes. XAFS spectrum was measured at room temperature.

3. Low-temperature Experiment Detail

3.1 Reaction system: Cu salt (0.5 mmol, 71.5 mg) was added to the schlenk tube cell in a glovebox beforehand. Then, 5.0 mL of THF was injected into the cell and the solution was stirred under

N_2 at – 78 °C for 20 minutes. Subsequently, RLi (2.5 mmol) was added into the system and stirred for 30 mins. As the last step, the liquid nitrogen was quickly added to reaction system, which would be frozen into solid immediately, and it was transferred into the XAFS cell with the protection of nitrogen gas.

3.2 Detection system (beamline): The detection system was cooled using cooled nitrogen gas. The Supplementary Fig. 5**.** was the picture of cell-holder used in the beamline. This hold connected with a liquid nitrogen cooled gas stream. The Supplementary Fig. 6 shows our idea for low-temperature system. We use a gas stream passing through the liquid nitrogen Dewar to cool the system. The temperature could be controlled by the tuning of the flow rate. The Supplementary Fig. 7 was the whole picture of experimental set-up in beamline.

DFT calculations

1 Complete reference for Gaussian 09

Gaussian 09, Revision A.2, Frisch, M. J.; Trucks, G. W.; Schlegel, H. B.; Scuseria, G. E.; Robb, M. A.; Cheeseman, J. R.; Scalmani, G.; Barone, V.; Mennucci, B.; Petersson, G. A.; Nakatsuji, H.; Caricato, M.; Li, X.; Hratchian, H. P.; Izmaylov, A. F.; Bloino, J.; Zheng, G.; Sonnenberg, J. L.; Hada, M.; Ehara, M.; Toyota, K.; Fukuda, R.; Hasegawa, J.; Ishida, M.; Nakajima, T.; Honda, Y.; Kitao, O.; Nakai, H.; Vreven, T.; Montgomery, Jr., J. A.; Peralta, J. E.; Ogliaro, F.; Bearpark, M.; Heyd, J. J.; Brothers, E.; Kudin, K. N.; Staroverov, V. N.; Kobayashi, R.; Normand, J.; Raghavachari, K.; Rendell, A.; Burant, J. C.; Iyengar, S. S.; Tomasi, J.; Cossi, M.; Rega, N.; Millam, N. J.; Klene, M.; Knox, J. E.; Cross, J. B.; Bakken, V.; Adamo, C.; Jaramillo, J.; Gomperts, R.; Stratmann, R. E.; Yazyev, O.; Austin, A. J.; Cammi, R.; Pomelli, C.; Ochterski, J. W.; Martin, R. L.; Morokuma, K.; Zakrzewski, V. G.; Voth, G. A.; Salvador, P.; Dannenberg, J. J.; Dapprich, S.; Daniels, A. D.; Farkas, Ö.; Foresman, J. B.; Ortiz, J. V.; Cioslowski, J.; Fox, D. J. Gaussian, Inc., Wallingford CT, **2013**.

2 B3LYP absolute calculation energies, enthalpies, and free energies.

All the DFT calculations were carried out with the GAUSSIAN 09 series of programs.

B3LYP functional[8] with 6-31G(d) basis set (SDD for Cu) was used for geometry optimizations. All the structure optimizations were performed in THF solvent with a SMD continuum solvation model.[9] Harmonic frequency calculations were performed for all stationary points to confirm them as a local minima or transition structures and to derive the thermo chemical corrections for the enthalpies and free energies. The energies given in this work are the B3LYP calculated Gibbs free energies.

Geometry	$E_{(THF)}$[1]	$H_{(corr\text{-}THF)}$[2]	$G_{(corr\text{-}THF)}$[3]	IF[4]
**$[Br\text{-}Cu\text{-}Br]^-$**	-5340.454850	0.007331	-0.027236	-
**THF**	-232.448360	0.123200	0.088879	-
**$[Br\text{-}Cu(THF)\text{-}Br]^-$**	-5572.903431	0.131174	0.072308	-
**$[^n$Bu-Cu-^{n}Bu$]^-$**	-513.1353274	0.255604	0.198092	-
**$[^n$Bu-Cu(THF)-^{n}Bu$]^-$**	-745.583958	0.380740	0.303081	-

[1]The electronic energy calculated by B3LYP in THF. [2]The thermal correction to enthalpy calculated by B3LYP in THF. [3]The thermal correction to Gibbs free energy calculated by B3LYP in THF. [4]The B3LYP calculated imaginary frequencies for the transition states.

3B3LYP geometries for all the optimized compounds and transition states.

$[Br\text{-}Cu\text{-}Br]^-$

Cu	0.00000000	0.00000000	0.00000000
Br	0.00000000	0.00000000	2.27331500
Br	0.00000000	0.00000000	-2.27331500

THF

C	1.16843700	-0.42818000	-0.13064600

O	0.00062400	-1.25396400	-0.00219400
C	-1.16721000	-0.42968100	0.13338400
C	-0.73446700	0.99545200	-0.22833500
C	0.73247200	0.99724800	0.22710200
H	1.95038500	-0.81981400	0.53037000
H	1.53903500	-0.48082300	-1.16560500
H	-1.95254800	-0.82337800	-0.52227200
H	-1.53148000	-0.48109500	1.17075100
H	-0.79732900	1.15131600	-1.31186600
H	-1.34517600	1.75933500	0.26227700
H	1.34194800	1.76091100	-0.26533800
H	0.79478800	1.15621700	1.31020500

[Br-Cu(THF)-Br]$^-$

Cu	-1.36729100	0.08499100	-0.00284100
Br	-1.17172000	2.35379100	-0.02404700
Br	-1.67874300	-2.17132300	0.02231700
C	3.05896300	-0.42606300	-1.16134300
O	2.24301500	-0.14519000	-0.01508000
C	3.04065800	-0.35581400	1.14910600
C	4.42622300	0.16408600	0.76054300
C	4.53782600	-0.29518000	-0.70923300
H	2.84557700	-1.43774200	-1.53430000
H	2.78168700	0.29018800	-1.94125900
H	2.57126100	0.18428400	1.97571100
H	3.08014000	-1.42771900	1.40685000
H	4.44286600	1.25757600	0.82741500
H	5.22549400	-0.23091000	1.39542300
H	5.09761100	0.41516300	-1.32478700
H	5.04689100	-1.26261500	-0.77591100

[^{n}Bu-Cu-^{n}Bu]$^-$

Cu	0.00001100	-0.00014100	0.00051300
C	-1.80931500	0.78332900	0.00046400
H	-1.91841700	1.45479300	0.87576700
H	-1.91803400	1.45537200	-0.87444500
C	-3.01268800	-0.18237100	-0.00014200
H	-2.96217600	-0.84988800	-0.87639700
H	-2.96264600	-0.85040400	0.87574600
C	-4.39792100	0.49234400	-0.00030200
H	-4.47302400	1.15132800	-0.87876100
H	-4.47346100	1.15086000	0.87847100
C	-5.57341600	-0.49336700	-0.00084500
H	-5.54369300	-1.14365500	0.88342700

H	-6.54234800	0.02211100	-0.00094300
H	-5.54326400	-1.14318500	-0.88544800
C	1.80938600	-0.78347700	0.00045400
H	1.91820200	-1.45546100	-0.87449100
H	1.91854800	-1.45496800	0.87573300
C	3.01263600	0.18237100	-0.00007700
H	2.96204000	0.84994300	-0.87628500
H	2.96251300	0.85034400	0.87585100
C	4.39797400	-0.49214400	-0.00029000
H	4.47314200	-1.15111000	-0.87875700
H	4.47363800	-1.15065200	0.87847800
C	5.57331200	0.49375400	-0.00088000
H	6.54232400	-0.02156900	-0.00103800
H	5.54300700	1.14358600	-0.88546800
H	5.54352600	1.14401700	0.88340800

[^{n}Bu-Cu(THF)-^{n}Bu]$^{-}$

C	2.17621800	1.29164700	-0.73568600
H	2.16619800	0.59605800	-1.59827100
H	2.45106500	2.27097900	-1.17719200
C	3.32617900	0.86199000	0.19891800
H	3.39489300	1.55148200	1.05710900
H	3.10716500	-0.12546600	0.63784500
C	4.71722600	0.78849900	-0.45981800
H	4.96109000	1.77266600	-0.88833000
H	4.67164800	0.09154400	-1.31044000
C	5.83908900	0.35620700	0.49320200
H	5.64097300	-0.64005800	0.91014700
H	6.81375700	0.31712800	-0.00995200
H	5.92976700	1.05207400	1.33783200
C	-1.44224100	1.51433300	0.81738300
H	-1.45749100	2.31375000	1.58565400
H	-1.66009500	0.58609900	1.38322600
C	-2.62729900	1.76461700	-0.13828300
H	-2.46607600	2.69473700	-0.70852600
H	-2.67580800	0.96526200	-0.89685300
C	-4.00852100	1.85933300	0.53806900
H	-3.98436200	2.66804000	1.28434000
H	-4.19222000	0.93272100	1.10313300
C	-5.16796400	2.10075200	-0.43724700
H	-6.13421900	2.16216200	0.07968400
H	-5.02823900	3.03799000	-0.99180300
H	-5.23765400	1.29124300	-1.17579300
Cu	0.36444600	1.39530100	0.03475900

C	0.23330300	-3.60506200	0.39647400
O	0.10368500	-2.93001100	-0.86441400
C	-1.26790900	-2.55867100	-1.08060900
C	-2.09026500	-3.35011300	-0.05900800
C	-1.10867600	-3.43800500	1.11968500
H	0.45626100	-4.66777700	0.21463200
H	1.07473100	-3.16945600	0.94654000
H	-1.38049000	-1.47612100	-0.92552300
H	-1.52858300	-2.78799200	-2.12049800
H	-3.03327000	-2.85686500	0.19558700
H	-2.31971600	-4.35217500	-0.44202400
H	-1.11994700	-2.50258000	1.69114000
H	-1.32262900	-4.26226000	1.80716300

Supplementary References:

1. Tsang KL, Lee CH, Jean YC, Dann TE, Chen JR, D'Amico KL, Oversluizen T. Wiggler x‐ray beamlines at Synchrotron Radiation Research Center. *Rev Sci Instrum* **66**, 1812-1814 (1995).
2. Nelson RC, Miller JT. An introduction to X-ray absorption spectroscopy and its in situ application to organometallic compounds and homogeneous catalysts. *Catal Sci Technol* **2**, 461-470 (2012).
3. Ravel B, Newville M. ATHENA, ARTEMIS, HEPHAESTUS: data analysis for X-ray absorption spectroscopy using IFEFFIT. *J Synchrotron Radiat* **12**, 537-541 (2005).
4. Yokoyama T, Satsukawa T, Ohta T. Anharmonic Interatomic Potentials of Metals and Metal Bromides Determined by EXAFS. *Jpn J Appl Phys* **28**, 1905-1908 (1989).
5. Briggs DN, Lawrence KH, Bell AT. An investigation of carbon-supported CuCl2/PdCl2 catalysts for diethyl carbonate synthesis. *Applied Catalysis A: General* **366**, 71-83 (2009).
6. Tranquada JM, Ingalls R. X-ray-absorption study of CuBr at high pressure. *Phys Rev B* **34**, 4267-4277 (1986).
7. Wang X-B, Wang L-S, Brown R, Schwerdtfeger P, Schröder D, Schwarz H. The electronic structure of CuCl2 and CuBr2 from anion photoelectron spectroscopy and ab initio calculations. *The Journal of Chemical Physics* **114**, 7388-7395 (2001).
8. Lee C, Yang W, Parr RG. Development of the Colle-Salvetti correlation-energy formula into a functional of the electron density. *Phys Rev B* **37**, 785-789 (1988).
9. Marenich AV, Cramer CJ, Truhlar DG. Universal solvation model based on solute electron density and on a continuum model of the solvent defined by the bulk dielectric constant and atomic surface tensions. *J Phys Chem B* **113**, 6378-6396 (2009).
